# Performance and safety of transverse scrotal vs transperineal AUS for PPUI: A retrospective cohort study

**DOI:** 10.1002/bco2.70027

**Published:** 2025-05-21

**Authors:** Christine R. Reus, Izabelle Brattås, Daniela Volz, Filip Sydén, Renata Zelic, Katarina Hallén Grufman, Lotta Renström Koskela

**Affiliations:** ^1^ Section of Urology, Department of Molecular Medicine and Surgery Karolinska Institute Stockholm Sweden; ^2^ Department of Pelvic Cancer Karolinska University Hospital Stockholm Sweden; ^3^ Section of Urology Södersjukhuset Stockholm Sweden; ^4^ Department of Surgery Capio St. Görans Hospital Stockholm Sweden

**Keywords:** (urinary incontinence), artificial urinary sphincter, pad‐test, post‐prostatectomy, quality of life

## Abstract

**Objectives:**

To primarily compare efficacy and safety of transverse scrotal (TS) versus transperineal (TP) artificial urinary sphincter (AUS) implantation for post‐prostatectomy urinary incontinence (PPUI). The AUS is the gold standard for managing severe refractory male SUI.

**Patients and Methods:**

This single‐centre, retrospective, cohort study, analyses 179 consecutive patients who underwent primary AUS implantation for PPUI between 2005 and 2018. Data on 24‐h pad weight tests (PWT), validated quality of life questionnaires (I‐QoL), surgical technique, related complications, salvage radiation and transcorporeal cuff placement (TC) were collected.

**Results:**

The TP approach was performed in 43 cases, whilst 136 patients underwent TS incision, of which 31 benefited from TC placement. The median PWT reduction was 458 g (320, 701) in the TP and 479 g (258, 745) in the TS group (p = 0.807). The median I‐QoL index increase was 40 (26, 52) in the TP and 48 (36, 60) in the TS group, showing a significant difference in favour of the TS group (p = 0.012). The overall postoperative infection rate was 3.9%, with a lower risk in the TS group (RR = 0.23, p = 0.049). Erosion occurred in 9.0% of patients, with a higher relative risk observed in the TS group (*RR = 1.34, p = 0.636*); however, we found that the TC patients (consisting of salvage radiation patients) in the TS group drove this higher risk. Mechanical failure and subsequent revision were lower in the TS cohort *(RR = 0.43, p = 0.004)* and *(RR = 0.42, p = 0.002),* respectively.

**Conclusion:**

TS and TP approaches resulted in similar improvements in continence but a greater increase in quality of life in the TS group. While post‐operative erosion rates and device survival were comparable, the TP group had higher rates of infection and mechanical failure, which may be relevant for surgical decision‐making.

## 1 INTRODUCTION

The AMS 800™ (Boston Scientific, Minnetonka, USA) is currently the ‘Gold Standard’ for severe male SUI refractory to conservative treatment. Both transperineal (TP), more frequently performed, and transverse scrotal (TS) approaches are routinely used, depending on the surgeon and institutional's preferences.

In the former technique, standard cuff placement involves a midline perineal incision to expose the proximal bulbar urethra and measure the cuff size with a cuff‐sizer. A lower abdominal incision allows for PRB placement in the pre‐peritoneal space. The pump is positioned in the scrotum. The device components are assembled, checked for leaks, and the AUS deactivated.

The TP approach is commonly used, allowing for more proximal urethral exposure and cuff positioning, potentially achieving better continence outcomes and reducing the need for additional interventions.

The latter technique was developed by Wilson in 2003, inspired by the inflatable penile prosthesis implantation, which presented the advantage of being swifter by using a single incision for the implantation of all AMS 800 components. The authors demonstrated the technique's feasibility and reported a ‘*zero pad*’ rate for continence outcomes of 66%, comparable to the TP approach.^1^ In 2010, Wilson refined the TS technique to allow more proximal occlusive cuff placement while maintaining a single incision. This modification improved deep bulbar exposure, facilitating simultaneous AUS and penile prosthesis implantation when necessary.^2^

However, long‐term continence outcomes remain controversial, owing to data paucity. Studies in favour of the TP are often low‐volume, retrospective, multicentric in nature. Kretschmer argued the TP was superior to the TS, based on a comparative six‐month follow‐up study including 99 TS implants, collecting data from 70.6% of patients. It showed an explantation rate of 8.6% for the TP compared to 19.2% for the TS cohort.^3^ Similarly, Yafi's study favoured the TP, as it found higher erosion, explantation and revision rates in the TS group.^4^

In contrast, Kenderci concluded that the TS technique was superior to the TP in a retrospective multicentre study including 22 patients, reporting a 14% revision rate due to erosion or PRB migration.^5^

Furthermore, Sotelo's study confirmed the non‐inferiority of the TS compared to the TS in a retrospective multi‐centre study including 83 AUS using the TS technique.^6^ Recent prospective and retrospective studies with longer follow‐ups found no significant difference in continence rates between the two approaches, suggesting that both techniques are effective.^7^, ^8^, ^9^ Additionally, a multicentre study conducted in 47 patients showed 4% erosion/infection rates, no mechanical failures, an improved pump placement. Consequently, the authors deemed the TS technique safe compared to the TP incision.^10^

Despite two decades of use, limited data exist on the long‐term efficacy and safety of TS versus TP AUS implantation for PPUI. A recent published abstract of a retrospective, single‐centre study comparing the above surgical approaches between 2000 and 2018 reported shorter operating times in the TS cohort, comparable continence outcomes of 75% in both groups (using the ‘*1 pad rate*’ definition) and no significant difference in complication rates in a cohort of 125 patients for an average follow‐up of 24 months. There are no published randomized controlled studies on the subject to date.^11^

This study aims to retrospectively compare the efficacy, safety and complication rates of TS versus TP primary AUS implantation in patients with PPUI, addressing the current gap in long‐term comparative data.

## 2 MATERIALS AND METHODS

### 2.1 Study design

This is a retrospective, comparative, single tertiary centre, cohort study of 179 consecutive men who underwent primary AUS implantation using the AMS 800™, by the same surgical team, between 2005 and 2018 at Karolinska University Hospital, Sweden.

### 2.2 Surgical technique, device components choice and settings

The TP technique was performed as described in the AMS 800™ Instruction for Use. Regarding the TS approach, we initially used the single incision approach, described by Wilson, later modified to a two‐incision approach (high transversal scrotal incision and lower abdominal incision), as described for the PRB placement in the TP approach. Cuff measurements were obtained using the cuff sizer provided in the AMS 800™ Accessory Kit™. The preferred PRB range was 60–71 cmH_2_O, with lower pressure ranges (51–60 cmH_2_O) in a few cases of post‐radiation therapy impaired urethra. Fluid volumes were chosen in accordance with the AMS 800™ IFU, using slightly larger volumes for bigger cuffs.

### 2.3 Inclusion and exclusion criteria

The study included men suffering from SUI after RP and/or following salvage RT for CaP who were refractory to conservative management (lifestyle changes, pelvic floor exercises and/or pharmacological treatment). Only patients who underwent primary AUS implantation were considered. Those with previously unsuccessful UI procedures prior to AUS surgery were included, on the condition that they continued to exhibit significant persistent bothersome SUI.

Secondary AUS implantations were excluded to avoid confounding factors caused by a secondary implantation of the device. Neurogenic cases were also excluded from this study. Two‐hundred and twenty‐one registered patients underwent primary AUS implantation between 2005 and 2018. Forty‐two patients were excluded: 3 women, 23 non‐PPUI‐related cases, 12 secondary AUS implantations and 4 patients who underwent a double cuff placement. Consequently, a total of 179 patients were included.

### 2.4 Data collection

We performed a retrospective data collection using the institution's EMR systems, adopted by Stockholm County since 2004. Baseline preoperative cohort characteristics were gathered, which included age, BMI, co‐morbidities, previous UIS, prior USS anastomotic stricture surgery, history of salvage RT and existing pre‐operative DOA.

Perioperatively, we recorded the AUS implantation technique used, TP or TS, as well as whether the OC was positioned transcorporally or not. AUS implant characteristics, such as OC size, amount of fluid in the PRB and PRB pressure ranges were collected, as was information on intraoperative AEs.

For functional outcome, data from the 24‐h PWT both pre‐operatively and at 3–6 months post‐AUS activation were gathered. Desirably, voiding diaries and 24‐h PWT should be completed on nonconsecutive days, a day whilst at home and a day when more physically active, for more accurate stress component evaluation.^12^, ^13^, ^14^ Standardized instructions on how to complete the PWT were provided for uniformity of data collection. Data on quality of life was collected from the validated *“Incontinence Quality of Life” (I‐QoL)* questionnaire, that have been handed out to our patients both prior to surgery and 3–6 months post‐AUS activation for the past twenty years in our department.^15^

Finally, we collected data on short‐ (<90 days) and long‐term post‐operative complications, as well as device survival.

### 2.5 Statistical analysis

All eligible patients were included in the study, thus, no sample size calculation was performed.

Numerical variables were summarized using mean and SD or median and interquartile range (IQR), where appropriate. Categorical variables were summarized as frequencies and compared using Pearson χ2 test. The equality of medians for continuous outcomes was compared using quantile regression. Missing values for pre‐ and/or post‐surgery PWT and I‐QoL were imputed using multiple imputation by chained equations, where we imputed 50 datasets under the assumption that, conditional on the observed data, the data were missing at random. The estimates from 50 multiply imputed data sets were combined using Rubin's rules. Risk ratios for binary outcomes, as well as their 95% confidence intervals (CIs) were estimated using a log binomial regression. The cumulative incidence function of device revision was estimated using the Aalen‐Johansen estimator. Cause‐specific hazard ratios (HRs) and 95% CIs were estimated using Cox proportional hazards regression. In the main analysis, we compared the association of TS vs TP treatment group and the outcome(s). In the secondary analysis, we compared TS and TC to TP. We further repeated all the analyses stratified by the previous RT, DOA, UIS and surgery for USS. All tests were 2‐sided, and the level of significance was set at p < 0.05.

All analyses were performed in Stata (version 17.0, StataCorp, College Station, Texas, USA).

### 2.6 Ethical considerations

The Regional Ethical Review Board granted ethical approval for this study.

## 3 RESULTS

### 3.1 Patient characteristics

The AMS 800™ was implanted using the TS technique in 136 patients, whilst the TP approach was used in 43. Trans‐corporeal cuff placement via TS incision was adopted in 31 out of 136 patients.

Cohort characteristics are presented in Table S1. The mean patient age at AUS implantation was 70.0 years (SD = 5.17), with a mean BMI of 26.5 (SD = 2.98). There was no difference between patients operated using TP or TS technique regarding age and BMI, even after further sub‐grouping TS into TS without TC cuff insertion vs TC alone. Additionally, five patients had a 51–60 cmH20 PRB. They underwent salvage radiotherapy, fragilizing their urethra, thereby warranting a lower pressure PRB, hoping to decrease erosion risks.

The history of salvage RT was identified in 21.8% (39/179) of the patients. There was a larger proportion of patients who received salvage RT in the TS group, 25.7% (35/136) as compared to the TP group, 9.3% (4/43). In the TS group, 85.7% (30/35) of the radiated patients had benefited from a TC placement. Furthermore, 16.2% (29/179) of patients had undergone previous (UIS), with a greater proportion of men having had previous UIS in the TP vs TS group. ProACT™ procedures were the most performed prior to primary AUS implantation, followed by bulking agents. Only three patients had undergone prior sling surgery. Additionally, 22.9% (41/179) of the patients had undergone USS prior to AUS insertion, 19.1% (26/136) in the TS and 34.8% (15/43) in the TP group. Finally, 41.9% (75/179) of patients were urodynamically diagnosed with DOA before surgery, with 43.4% (59/136) in the TS and 37.2% (16/43) in the TP group.

### 3.2 Implanted AUS characteristics

AUS characteristics are described in Table S1. The most frequently used cuff size was 4.5 cm in all groups, with a larger proportion of bigger cuff sizes in the TP and TC group. The majority of implanted PRBs, 94.4% (168/178), were in the pressure range 61–70 cmH_2_O. Seven of the implanted PRBs were in the 51–60 cmH_2_O range, five of which were used in the TP group and two in the TC group. The volumes used to fill the PRB were 24 ml in patients operated with the TS approach but varied between 21 and 28 ml in the TP group, with a median of 24 ml. Larger volumes were used in cuffs ≥ 4.5 cm.

### 3.3 Continence outcomes

Out of 179 patients, 155 patients (86.6%) had correctly completed both pre‐ and post‐operative 24‐h PWT. An overall median difference between the pre‐ and post‐operative 24‐h PWT of 474.0 g (IQR 274, 743) showed a clinically meaningful decrease in UI following AUS implantation, with a median PWT of 7 g (IQR 0, 25) after activation (Table 1A).

**TABLE 1:** Functional and qualitative outcomes. **A.** Distribution of functional and qualitative outcomes for different surgical approaches. Complete case analysis, adjusted for pre‐surgery measurements of the same outcome. **B.** Differences in functional and qualitative outcomes for different surgical approaches. Imputaded data analysis.

Table 1A	Total (N = 179)			TP (N = 43)			TS including TC (N = 136)			TS excluding TC (N = 105)			TC (N = 31)		
	N	Median	IQR	N	Median	IQR	N	Median	IQR	N	Median	IQR	N	Median	IQR
**24 h pad weight test**															
Pre‐surgery	155	484,00	295.00, 767.00	35	474,00	323.00, 750.00	120	488,50	281.50, 767.00	97	450,00	280.00, 714.00	23	630,00	350.00, 963.00
Post‐surgery	155	7,00	0.00, 25.00	35	8,00	0.00, 25.00	120	7,00	0.00, 25.50	97	7,00	0.00, 25.00	23	12,00	0.00, 27.00
Diff. post‐ and pre‐surgery	155	−474,00	−743.00, −274.00	35	−458,00	−701.00, −320.00	120	−478,50	−745.00, −258.00	97	−442,00	−683.00, −243.00	23	−590,00	−963.00, −350.00
**I‐QoL Index**															
Pre‐surgery	97	34,00	22.00, 51.00	26	29,00	22.00, 48.00	71	36,00	22.00, 52.00	58	38,50	23.00, 53.00	13	34,00	13.00, 45.00
Post‐surgery	97	86,00	74.00, 94.00	26	76,00	64.00, 86.00	71	88,00	76.00, 95.00	58	88,50	75.00, 97.00	13	88,00	86.00, 93.00
Diff. post‐ and pre‐surgery	97	47,00	31.00, 58.00	26	40,00	26.00, 52.00	71	48,00	36.00, 60.00	58	47,50	35.00, 59.00	13	52,00	44.00, 71.00

Table 1B	Crude difference of medians				Adjusted difference of medians*			
	Diff	95% CIs		p‐value	Diff	95% CIs		p‐value
**Difference between post‐ and pre‐surgery 24 h PWT**								
TS including TC vs TP	0,64	−168,88	170,16	0,994	−1,30	−11,80	9,19	0,807
TS excluding TC vs TP	28,84	−151,97	209,65	0,753	−1,93	−12,86	9,00	0,727
TC vs TP	−98,24	−340,07	143,59	0,424	3,50	−10,92	17,92	0,632
**Difference between post‐ and pre‐surgery I‐QoL index**								
TS including TC vs TP	8,46	−2,34	19,26	0,123	9,70	2,16	17,23	***0,012***
TS excluding TC vs TP	6,94	−4,45	18,33	0,230	8,60	0,73	16,48	***0,033***
TC vs TP	17,82	2,87	32,77	***0,020***	10,96	0,91	21,02	***0,033***

**TP:** Transperineal.

**TS:** Transscrotal.

**TC:** Transcorporeal cuff placement in patients operated with a transscrotal incision.

Median 24 h‐pad weight test before and after surgery as well as the median reduction i leakage, complete case analysis (A). There was no difference in medians between patients operated with a transscrotal incision as compared to those operated with the transperineal approach, imputated data (B). Furthermore, no difference was seen when sub‐grouping in regard to patients in the transscrotal group that had received a transcorporeal cuff placement (B). Median pre‐ and post‐operative I‐QoL index as well as median increase in I‐QoL index, complete case analysis (A). For the I‐QoL index there was a difference in medians between patients operated with a transscrotal incision as compared to those operated with the transperineal approach, imputated data (B). Furthermore, this difference was seen when sub‐grouping in regard to patients in the transscrotal group that had received a transcorporeal cuff (B).

Both pre‐ and post‐operative 24‐h PWT were successfully completed by 81.4% (35/43) in the TP group and 88.2% (120/136) of patients in the TS group, of which 21.9% (23/105) had received a TCOC. We found no difference in the median PW reduction between the TS vs TP groups (adjusted difference in medians −1.30(95% CIs: −11.8, 9.2);*p = (0.807)* as shown in Table 1B. Similarly, there was no difference in the median PW reduction in the TC vs TP group, 3.50 (95% CIs: −10.92, 17.92); *p = (0.632)* (Table 1B).

**FIGURE 1:**
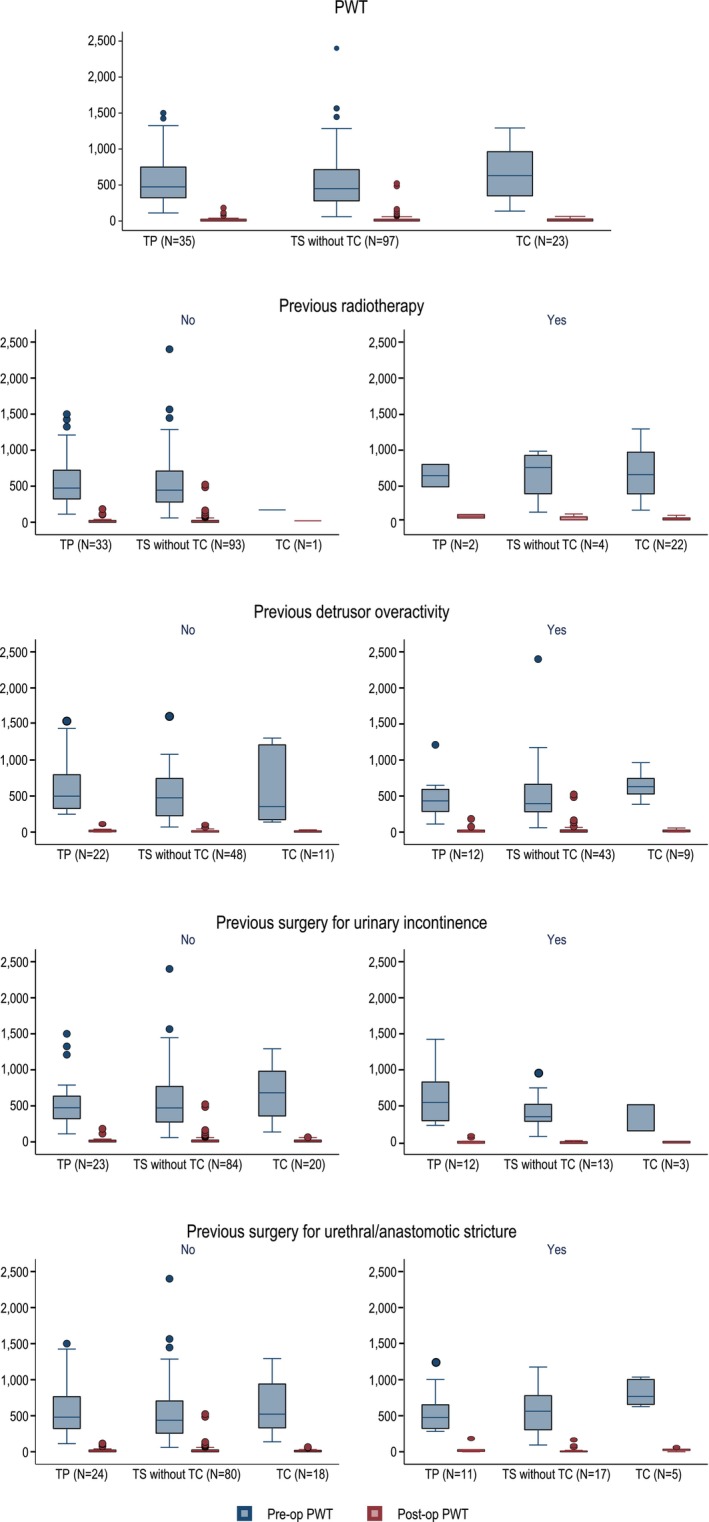
**Functional outcome.** Pre‐ and post‐operative 24 h‐pad weight test in each treatment group (transperineal (TP), transscrotal (TS) excluding transcorporeal cuff placement and patients with a transcorporeal cuff placement (TC)) as well as stratified by concomitant detrusor overactivity, previous radiation therapy, previous surgery for urinary incontinence and surgery for urethral stricture. No difference was seen in pad weight reduction between the TS and TC vs TP group in any of the analyses.

Distributions of the pre‐ and post‐operative 24‐h PWT, as well as of the difference between the two measurements in each treatment group stratified by concomitant DOA, previous RT, prior UIS and USS are reported in Table S2. We found no difference in PW reduction between the TS vs TP group, and the TC vs TP group in the stratified analysis (Figure 1 and Table S3).

### 3.4 Qualitative outcomes at 3–6 months post‐device activation

Only 54.2% (97/179) of patients had correctly filled in the I‐QoL survey both pre‐ and post‐operatively. We found an overall median pre‐ and post‐device activation I‐QoL index increase of 47 points (IQR 31, 58), Table 1A.

Both pre‐ and post‐operative I‐QoL surveys were correctly completed by 60.5% (26/43) of patients in the TP group and 52.2% (71/136) of patients in the TS group, of which 22.8% (31/136) received a TC occlusive cuff. We observed higher median pre‐ and post‐operative I‐QoL index in the patients operated with a TS approach 48 (IQR 36, 60) compared to the TP group 40 (IQR 26, 52) (Table 1A), showing a difference in the median I‐QoL index increase between the two groups (adjusted difference in medians [970 (95% CIs: 2.16, 17.23); *p = 0,012*] as presented in Table 1B. Similarly, we found a significant difference in the median I‐QoL index increase when we compared TC vs TP groups (Table 1B).

**FIGURE 2:**
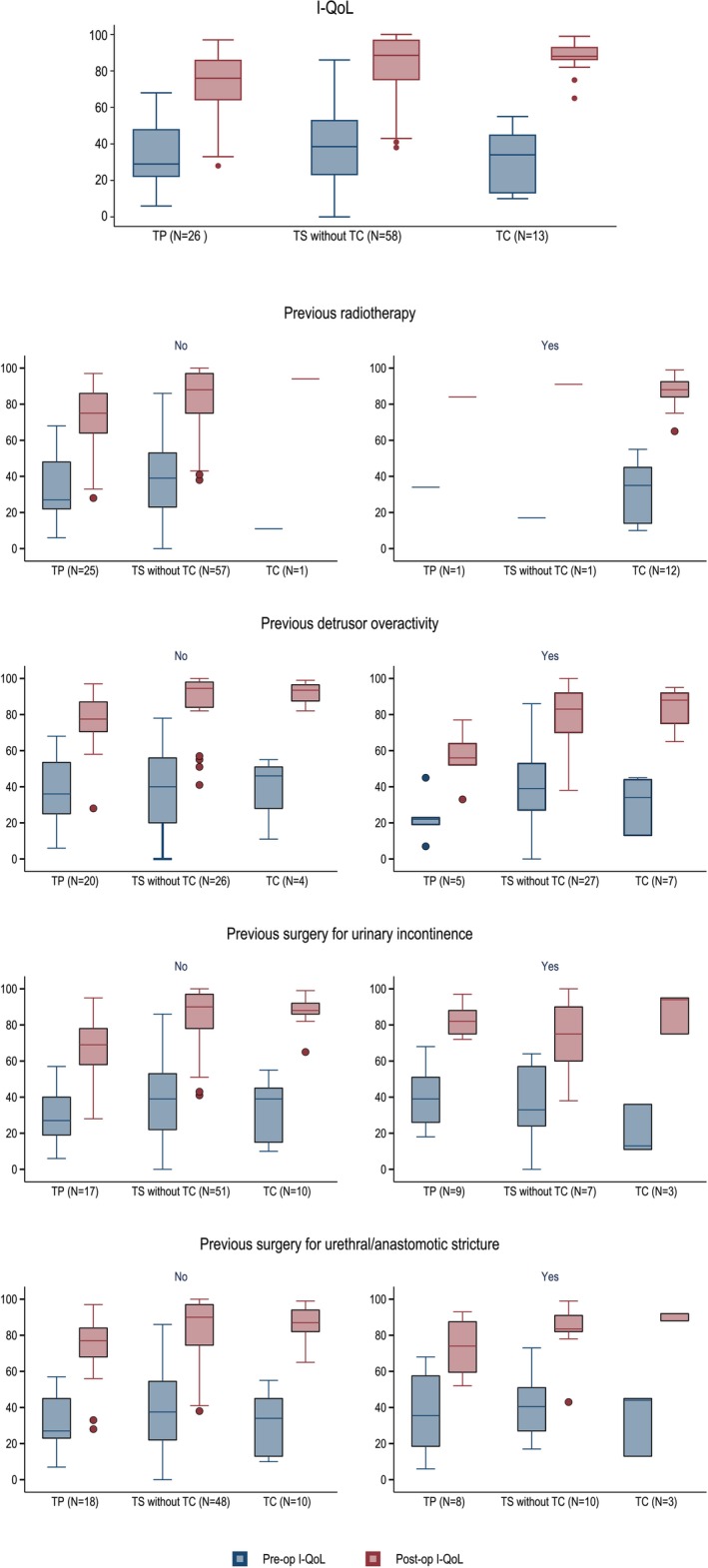
**Qualitative outcome measurement.** Pre‐ and post‐operative I‐QoL index in each treatment group (transperineal (TP), transscrotal (TS), excluding transcorporeal cuff placement and patients with a transcorporeal cuff placement (TC)) as well as stratified by concomitant detrusor overactivity, previous radiation therapy, previous surgery for urinary incontinence and surgery for urethral stricture. Overall there was a significant difference in increase of I‐QoL between the TS vs TP and the TC vs TP group. In the stratified analysis, a benefit was found in the TS group compared to the TP group for patients with no DOA, no previous surgery for urinary incontinence and no previous surgery for urethral strictures.

Distributions of the pre‐ and post‐operative I‐QoL index, as well as of the difference between the two measurements in each treatment group in the stratified analysis, are presented in Figure 2 and Table S2. Overall, there was no difference in the median I‐QoL index increase in the TS vs TP group in the stratified analysis, except for a higher median I‐QoL index increase for TS patients without DOA, previous s UIS and USS (Table S3).

### 3.5 Safety outcomes

#### 3.5.1 Early perioperative and postoperative adverse events

Overall, 12.3% (22/179) of early adverse events occurred within 90 days. The most reported AEs were observed for Clavien‐Dindo Class I‐II (non‐serious AEs) in all three groups, 6.9% (3/43) in the TP, and 5.1% (7/136) in the TS (including TC cuff placements). When separating TC patients from the TS group 9.7% (3/31) occurred in the TC group and 3.8% (4/105) in the rest of the TS group. However, TP procedures included higher Class III a,b (serious AEs) occurrences, 16.3% (7/43), as opposed to 3.8% (4/105) in the TS group and 3.2% (1/31) in the TC group. In the TP group, one patient had an intraoperative urethral injury and therefore, no device was placed following primary repair of the injury. The results are summarized in Table 2.

**TABLE 2:** Early complications (<90 days) according to Clavien‐Dindo classification.

Clavien‐Dindo classification	Symptoms	Management	AUS implantation technique n (%)			
			TP (n = 43)	TS (n = 105)	TC (n = 31)	TOTAL (n = 179)
**I**	Pain, hematoma, urinary retention, superficial wound infection	Analgesia, conservative management, urethral catheterization, antibiotics	2(4.7)	4(3.8)	3(9.7)	9(5.0)
**II**	Urinary tract infection	Antibiotics	1(2.3)	0(0.0)	0(0.0)	1(0.6)
**IIIa**	Bleeding	Re‐operation with diathermia	0(0.0)	1(1.0)	0(0.0)	1(0.6)
**IIIb**	Migration of device components	Revision	2(4.7)	1(1.0)	0(0.0)	3(1.7)
	Intra‐operative urethral injury	Abandon procedure	1(2.3)	0(0.0)	0(0.0)	1(0.6)
	Implant infection	Explantation	4(9.3)	1(1.0)	0(0.0)	5(2.8)
	Urinary retention	Revision (cuff re‐sizing)	0(0.0)	0(0.0)	1(3.2)	1(0.6)
	Erosion	Explantation	0(0.0)	1(1.0)	0(0.0)	1(0.6)
**IV‐V**			0(0.0)	0(0.0)	0(0.0)	0(0.0)
**TOTAL**			**10(23.3)**	**8(7.6)**	**4(12.9)**	**22(12.3)**

#### 3.5.2 Serious adverse events

Fifty‐four long‐term AEs have been registered in 178 patients (30.3%) over a 17‐year period, leading to 63 re‐interventions, 38 revisions (of which 36 were secondary to mechanical failure and 25 to device explantations. In total, seven patients had an implant infection, with three occurring more than 90 days post‐implantation. A total of 16 patients had an erosion of their implant, one of which occurred less than 90 days from device implantation.

Overall, 3.9% (7/178) of patients had a post‐operative *infectio*n, whilst *erosion* occurred in 9.0% (16/178) of patients and 20.2% (36/178) had a *mechanical failure* of the implant (Table 3). As expected, the risk of *revision* mirrored the risk for mechanical failure, 21.3% (38/178). When compared to the patients in the TP group, patients in the TS group had a lower risk of *infection* (RR = 0.23, [95% CIs: 0.05, 0.99] p = 0.049), *explantation* (RR = 0.79, [95% CIs: 0.36, 0.1,77] p = 0.573), *mechanical failure* (RR = 0.43, [95% CIs: 0.25, 0.76] p = 0.004) and *revision* (RR = 0.42, [95% CIs 0.25, 0.73] p = 0.002), and a higher risk of *erosion* (RR = 1.34, [95% CIs: 0.40, 4.47] p = 0.636) (Table 3). However, when comparing TS and TC to the TP group, we found that the TC group was driving the higher risk of erosion (RR = 3.16, [95% CIs: 0.89, 11.26] p = 0.076) and that the TC group also had a higher risk of explanation, albeit with wide CIs (RR = 1.55, [95% CIs: 0.63, 3.82] p = 0.342) (Table 3).

**TABLE 3:** Overall rates of infection, erosion, mechanical failure, revision and explantation for different surgical approaches (A). Risk ratios and risk differences between the groups (B).

A	Total		TP		TS including TC		TS excluding TC		TC	
	N = 178*		N = 42*		N = 136		N = 105		N = 31	
	N	%	N	%	N	%	N	%	N	%
Infection	7	3.93	4	9.52	3	2.21	3	2.86	0	0.00
Erosion	16	8.99	3	7.14	13	9.56	6	5.71	7	22.58
Mechanical failure	36	20.22	15	35.71	21	15.44	16	15.24	5	16.13
Revision	38	21.35	16	38.10	22	16.18	17	16.19	5	16.13
Explantation	25	14.04	7	16.67	18	13.24	10	9.52	8	25.81

B	Risk ratios				Risk difference			
	RR	95% CIs		p‐value	RD	95% CIs		p‐value
Infection								
TS including TC vs TP	0,23	0,05	0,99	***0,049***	−7,32	−16,53	1,90	0,120
TS excluding TC vs TP	0,30	0,07	1,28	0,104	−6,67	−16,10	2,77	0,166
TC vs TP	1,00				0,00	0,00	0,00	
Erosion								
TS including TC vs TP	1,34	0,40	4,47	0,636	2,42	−6,81	11,64	0,608
TS excluding TC vs TP	0,80	0,21	3,05	0,744	−1,43	−10,39	7,54	0,755
TC vs TP	3,16	0,89	11,26	0,076	15,44	−1,21	32,09	0,069
Mechanical failure								
TS including TC vs TP	0,43	0,25	0,76	***0,004***	−20,27	−35,99	−4,56	***0,011***
TS excluding TC vs TP	0,43	0,23	0,78	***0,006***	−20,48	−36,52	−4,44	***0,012***
TC vs TP	0,45	0,18	1,11	0,083	−19,59	−39,02	−0,15	***0,048***
Revision								
TS including TC vs TP	0,42	0,25	0,73	***0,002***	−21,92	−37,86	−5,98	***0,007***
TS excluding TC vs TP	0,43	0,24	0,76	***0,004***	−21,90	−38,19	−5,62	***0,008***
TC vs TP	0,42	0,17	1,03	0,059	−21,97	−41,54	−2,39	***0,028***
Explantation								
TS including TC vs TP	0,79	0,36	1,77	0,573	−3,43	−16,06	9,20	0,594
TS excluding TC vs TP	0,57	0,23	1,40	0,221	−7,14	−19,73	5,45	0,266
TC vs TP	1,55	0,63	3,82	0,342	9,14	−9,95	28,23	0,348

**TP:** Transperineal.

**TS:** Transscrotal.

**TC:** Transcorporeal cuff placement in patients operated with a transscrotal incision.

* In one patient operated with the TP approach no implant was placed due to an intraoperative injury to the urethra, thus excluded from the postoperative analysis.

In the stratified analysis for DOA, previous RT, previous UIS and USS, differences in the RR for TS vs TP within the strata were only seen for mechanical failure and revisions (Table S4).

*Mechanical failure* was common, with the most frequently registered device failure being cuff‐related (40.5%), followed by PBR‐related failure in 29.7% of the cases. Defects in the tubing accounted for 13.5%, and pump failure for 10.8%. The cause for mechanical failure was unspecified in 5.4%.

**FIGURE 3:**
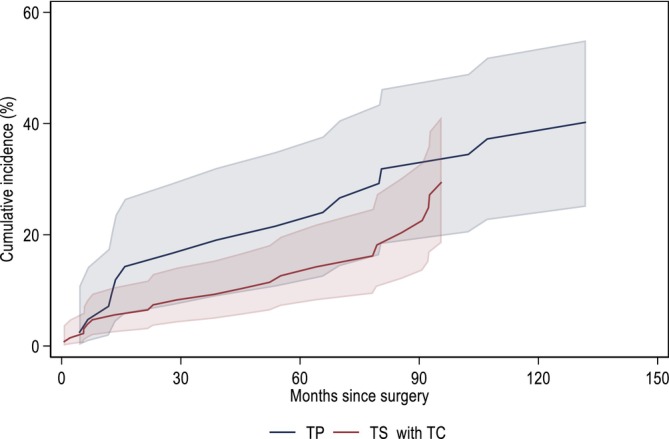
**Cumulative incidence of device revision**. Being implanted via TS approach was associated with a lower hazard of device revision, with, however, wide confidence intervals (HR = 0.63, 95% CIs: 0.32, 1.22, p = 0.154).

The cumulative incidence of *device revision* among subjects treated with TP and TS is presented in Figure 3. Being implanted via the TS approach was associated with a lower hazard of device revision, with, however, wide confidence intervals (HR = 0.63, 95% CIs: 0.32, 1.22). There were no obvious differences in the effect of treatment on the device survival in the stratified analysis (data not shown).

## 4 DISCUSSION

To our knowledge, this is the largest retrospective cohort study comparing the performance of primary TS versus TP AUS implantation in men with PPUI using the 24‐h PWT and safety outcomes. Additionally, this study explores whether the performance of TS vs TP AUS is modified by salvage RT, TC placement, pre‐operative DOA, previous UIS or previous USS or anastomotic stricture surgery.

### 4.1 Surgical technique

Although AUS implantation via TP incision is used in our institution, the preferred approach is the TS technique, using the enhanced method described by Wilson et al, as referred above, to achieve more proximal placement of the occlusive cuff. Furthermore, we find this technique quicker to perform. We perform the TP approach as classically described in textbooks and in the AMS 800™ operating manual (see annex 1).^16^ Historically, our institution has initially performed the TP technique and introduced the TS approach in 2010, using at first a single incision and later moved to a two‐incision technique to reduce PRB migration. This practice would account for the higher revision and mechanical failure observed in this study for the TP cohort compared to the TS, since TP devices remained in situ for a longer period.

### 4.2 Performance outcomes

We found no difference between both groups in terms of performance outcomes, using the 24‐h PWT as outcome measure, in line with previously published series.^1^, ^2^ It is noteworthy to mention that different continence definitions were used to define performance outcomes in these studies. In 2019, singal presented a single‐centre, retrospective cohort study from 2000 to 2018, which included 125 patients, of which 64% (80/125) were fitted with a TS AUS and 36% (45/125) with TP AUS. Their results showed similar social continence (using a ‘*one pad per day*’ definition) and safety outcomes.^11^ Another study published 10 years ago, analysing 21 men with a total of 27 AUS, 12 TP and 15 TS, showed no difference in terms of long‐term ‘completely dry’ rates (TS vs TP: 66.7% vs 50%, P = 0.767).^11^ However, the authors of a third study including 126 AUS, 63 implanted using the TS and 63 using the TP technique, concluded that the TP approach provided higher dry rates compared to the TS,^17^ a fact that could be explained by a more distal cuff placement in the TS group, as often performed in the earlier days of TS approaches.

Our study is in keeping with a recent French retrospective multicentric study published earlier this year by Bernard, including 1179 men who benefited from a primary AUS implantation for PPUI. The authors reported 762 men in the TP arm and 417 in the TS. Both cohorts had similar baseline characteristics, comparable overall reintervention‐free device survival rates. Interestingly, higher ‘dry’ continence rates were reported in the TS group (54%) compared to the TP (42%).^7^

### 4.3 Qualitative outcomes

Similarly, we observed a clinically significant QoL improvement following AUS implantation, with a higher median increase in I‐QoL in the TS technique. Since there are no prior studies comparing TS versus TP qualitative outcomes, we found our results to be in keeping with the overall findings published in Van der Aa's systematic review, the Mayo Clinic 2016 retrospective study and the MASTER Trial.^18^, ^19^, ^20^ We must bear in mind that, owing to the lack of standardized use of validated questionnaires, the short‐to mid‐term nature of our results, as well as the recent collective awareness of the importance of QoL, data comparison remains challenging.

### 4.4 Safety outcomes

Short‐term AEs were similar in both TS and TP groups, although higher rates of serious AEs were observed in the latter. Our results were comparable to those published in the MASTER trial regarding the TP group. Interestingly, none of the studies comparing TS and TP techniques delved into detailed AEs description, so we cannot draw any comparisons to other studies.

Overall, the post‐operative infection rate of 3.9% observed was lower than the previously reported 8.5% pooled analysis of AUS outcomes,^19^ with an increased risk for the TP approach. Singal also published slightly higher rates in the TP vs TS group, albeit not statistically significant (15.6% versus 11.3%, p = 0.489).^11^ Similar results were also found in Henry (20% for the TS and 33.3% for the TP group, p = 0.617).^10^ This fact could perhaps be partially explained by the pre‐, intra‐ and post‐operative antibiotic prophylaxis regime previously described, combined with the systematic use of Inhibizone after 2008 and the strictly limited perioperative theatre traffic observed in our institution, although these elements remain unproven. Indeed, very few studies elaborate on the antibiotics used,^19^ making it very difficult once again to draw any conclusion.

Interestingly, most studies comparing TS and TP techniques have shorter follow‐ups; for instance, Singal et al. report 2.2% mechanical failure rates for TP and 1.3% for TS (p = 0.678) for ‘an average follow‐up interval’ of 24.03 months.^11^ Since mechanical failure rates increase with the age of the device, and because this study evaluates the results up to 13 years post‐implantation, these could attest for the high percentages observed. Furthermore, as mentioned previously, the TP group would have been implanted longer than the TS AUS for historical reasons in our cohort.

However, regarding erosion rates, we showed similar rates in both techniques, despite a higher percentage of patients with a history of salvage RT in the TS group. This could be attributed to the fact that most of the radiated patients in the TS group had received a TC cuff. Also, our reported overall revision rates, with an increased risk in the TP group, and our overall explantation rates, with an increased risk in the TP group, were all in line with reported outcomes in the literature.^19^

Our study is one of the very few studies comparing TS versus TP device survival, which showed no difference between both incision types, in line with results from Henry,^10^ Bernard^7^ and Singal.^11^ We also accounted for competing events, which are common in these men, by using Aalen–Johansen estimator instead of Kaplan–Meier estimator to obtain the cumulative incidence of device failure.

Furthermore, we found no differences in functional outcomes in terms of 24‐h pad weight reduction between the TS and TC compared with the TP in the stratified analysis for DOA, salvage RT, previous UIS and prior USS. However, slight differences in the relative risk analysis in the same stratified group were observed, although owing to the low precision of estimates, we are unable to exclude happenstance. However, our study results are in line with the Mayo Clinic findings for the TP group, suggesting that salvage RT does not decrease continence rate. These results could be related to the constant improvement of radiation techniques and dose refinement, inducing less tissue damage. Additionally, subgroup analysis for RT in patients with TP AUS has been extensively published.^21^, ^22^, ^23^, ^24^, ^25^ Nevertheless, to date, such detailed stratified analysis has not yet been conducted for TS versus TP techniques, and therefore, our results cannot be compared to any available data in the literature.

### 4.5 Strengths and limitations

One strength of this study resides in its analysis of comparative qualitative outcomes in both groups, and the fact that our institution is a tertiary centre contributes to reduce selection bias. However, the main drawback of our study is its retrospective nature and its limited cohort number, an inevitable fact shared with most studies on the subject, since AUS implantation constitutes a minority of urological procedures performed by a few surgeons worldwide. Given the limited cohort size, the study may possibly be underpowered for analysis of rare outcomes in the exploratory analysis.

### 4.6 Future perspectives

In an era of evidence‐based medicine, retrospective studies are often the first step for data gathering. AUS surgery is only performed in a few highly specialized centres worldwide, and therefore, attaining statistically significant patient cohorts over the years is challenging. The way forward to improve long‐term performance and safety knowledge on the device and its implantation techniques is to carry out prospective multi‐centric studies, as mentioned in the French study mentioned above.^7^ The SATURN trial has completed its inclusion process, and follow‐up data collection is ongoing.^26^ This multicentric European study will shed light on long‐term results comparing both techniques in a larger cohort. Future research should focus on multicentre collaborations to increase cohort size and standardize outcome measures.

## 5 CONCLUSION

In summary, this retrospective single‐centre cohort study showed no difference in performance and qualitative outcomes when comparing transscrotal and transperineal techniques, in line with other published data. Furthermore, analysis stratified by history of RT, previous UIS, urethral stricture and DOA indicated no effect modification by these variables. Nonetheless, we found an increased infection risk, increased risk for mechanical failures and a higher revision risk in the transperineal group. However, erosion risks and device survival were similar in both techniques.

There is a need to improve long‐term patient follow‐up and to implement multicentre prospective studies with the aim to collect more reliable and comparable data, using validated patient‐reported outcome measures to improve long‐term patient functional and safety outcomes.

## AUTHOR CONTRIBUTIONS

Christine R. Reus had full access to the presented data in this study and takes responsibility for the integrity and accuracy of the data analysis. *Study concept and design*: Renström‐Koskela, Hallén Grufman. *Data acquisition*: Reus, Brattås, Sydén and Volz. *Drafting of the manuscript*: Reus, Brattås. *Critical revision of the manuscript for important intellectual contents*: all authors. *Statistical analysis*: Renata Zelic. *Supervision*: Renström Koskela.

## CONFLICT OF INTEREST STATEMENT

Christine R. Reus and Lotta Renström‐Koskela are Speakers for Boston Scientific. The remaining contributing authors have no conflicts of interest, including specific financial interests, relationships and affiliations pertaining to the above subject or materials discussed in this article.

## Supporting information


**Table S1.** Cohort and AUS characteristics.

**Table S2.** **Functional and qualitative outcomes, stratified analysis.** Distributions of the pre‐ and post‐operative 24‐h pad weight test and I‐QoL index as well as of the difference between the pre‐ and post‐operative measurements in each treatment group stratified by previous radiotherapy, concomitant detrusor overactivity, previous surgery for urinary incontinence and previous surgery for strictures. Complete case analysis.

**Table S3.** **Difference in medians regarding changes in 24‐h pad weight test and I‐QoL.** There was no difference in medians for the reduction in 24‐h pad weight test between surgical groups when stratifying for previous radiotherapy, detrusor overactivity, surgery for urinary incontinence or surgery for urethral or anastomotic stricture. For patients with no concomitant detrusor overactivity, previous surgery for urinary incontinence or surgery for urethral or anastomotic stricture, we found a higher increase in I‐QoL index after implantation in patients benefitting from a transscrotal approach compared to patients operated with a transperineal approach. Imputed data analysis.

**Table S4.** **Relative risks for serious adverse device events, stratified analysis.** Relative risk for serious adverse device events depending on different surgical techniques, stratified for previous radiation therapy, previous surgery for incontinence, previous surgery for urethral/anastomotic stricture and detrusor overactivity. Empty cells show that no patient within the strata had the complication of interest.
