# Supplementary material for: Performance and safety of transverse scrotal vs transperineal AUS for PPUI: A retrospective cohort study
Source: BJUI Compass. 2025 May 21;6(5):e70027. doi: 10.1002/bco2.70027 (PMC12094818; doi:10.1002/bco2.70027)
Supplement: Supplementary file 1 — Table S1. Cohort and AUS characteristics. [file BCO2-6-e70027-s003.pdf]

Supplemental table 1

Cohort and AUS characteristics

Patient characteristics	Total N=179		TP N=43		TS including TC N=136		TS excluding TC N=105		TC N=31	
Age, years (mean, SD)	70,0	5.17	68,6	4.49	70,5	5.31	70,4	5.43	70,7	4.93
BMI, kg/m2 (mean, SD)	26,5	2.98	26,1	2.87	26,6	3.02	26,4	3.00	27,4	2.99
**Patient characteristics**	**N**	**%**	**N**	**%**	**N**	**%**	**N**	**%**	**N**	**%**
DOA	75	41.90	16	37.21	59	43.38	45	42.86	14	45.16
Previous UIS	29	16.20	13	30.23	16	11.76	13	12.38	3	9.68
Previous USS	41	22.90	15	34.88	26	19.12	19	18.10	7	22.58
Previous RT	39	21.79	4	9.30	35	25.74	5	4.76	30	96.77
**AUS Characteristics***	**N***	**%**	**N***	**%**	**N**	**%**	**N**	**%**	**N**	**%**
**Cuff size**										
- 3.5 cm	5	2.81	0	0.00	5	3.68	5	4.76	0	0.00
- 4.0 cm	52	29.21	8	19.05	44	32.35	40	38.10	4	12.90
- 4.5 cm	78	43.82	19	45.24	59	43.38	46	43.81	13	41.94
- 5.0 cm	37	20.79	12	28.57	25	18.38	13	12.38	12	38.71
- 5.5 cm	3	1.69	1	2.38	2	1.47	0	0.00	2	6.45
**PRB pressure**										
- 51-60 cmH2O	7	3,93	5	11.90	2	1.47	0	0	2	6.45
- 61-70 cm H2O	168	94,38	36	85.71	132	97.06	103	98.1	29	93.54
**PRB volume**										
- 21-22 ml	9	5,06	9	18,75	0	0.00	0	0.0	0	0.0
- 23 ml	15	8,43	15	31,25	0	0.00	0	0.0	0	0.0
- 24 ml	145	81.46	13	30.95	132	97.06	103	98.10	29	93.55
- 25-28 ml	2	1,12	2	4,17	0	0.00	0	0.0	0	0.0

*In one patient operated with the TP approach no implant was placed due to an intraoperative injurie to the urehra.

TP: Transperineal
TS: Transscrotal
TC: Transcorporeal
DOA: Detrusor over activity
UIS: Urinary incontinence surgery
USS: Urethral/anastomotic stricture surgery
RT: Radio therapy
AUS: Artificial urinary sphincter
PRB: Pressure regulating balloon
