# Supplementary material for: Performance and safety of transverse scrotal vs transperineal AUS for PPUI: A retrospective cohort study
Source: BJUI Compass. 2025 May 21;6(5):e70027. doi: 10.1002/bco2.70027 (PMC12094818; doi:10.1002/bco2.70027)
Supplement: Supplementary file 2 — Table S2. Functional and qualitative outcomes, stratified analysis. Distributions of the pre‐ and post‐operative 24‐h pad weight test and I‐QoL index as well as of the difference between the pre‐ and post‐operative measurements in each treatment group stratified by previous radiotherapy, concomitant detrusor overactivity, previous surgery for urinary incontinence and previous surgery for strictures. Complete case analysis. [file BCO2-6-e70027-s004.pdf]

**Supplement table 2**

Distribution of functional and qualitative outcomes for different surgical approaches, stratified analysis.
Complete case analysis, adjusted for pre-surgery measurements of the same outcome.

| | Total | | | TP | | | TS with TC | | | TS without TC | | | TC | | |
|---|---|---|---|---|---|---|---|---|---|---|---|---|---|---|---|
| | N | Median | 95% CI | N | Median | 95% CI | N | Median | 95% CI | N | Median | 95% CI | N | Median | 95% CI |
| **No previous radiotherapy** | | | | | | | | | | | | | | | |
| Pre-surgery 24h PWT | 127 | 448 | 282.00, 714.00 | 33 | 474,00 | 323.00, 722.00 | 94 | 445,00 | 280.00, 710.00 | 93 | 445,00 | 280.00, 710.00 | 1 | 171,00 | 171.00, 171.00 |
| Post-surgery 24h PWT | 127 | 7 | 0.00, 24.00 | 33 | 7,00 | 0.00, 23.00 | 94 | 7,00 | 0.00, 25.00 | 93 | 7,00 | 0.00, 25.00 | 1 | 19,00 | 19.00, 19.00 |
| Diff. post- and pre-surgery 24h PWT | 127 | -441 | -675.00, -255.00 | 33 | -458,00 | -675.00, -320.00 | 94 | -441,00 | -665.00, -243.00 | 93 | -441,00 | -674.00, -243.00 | 1 | -152,00 | -152.00, -152.00 |
| Pre-surgery I-QoL | 83 | 36 | 22.00, 53.00 | 25 | 27,00 | 22.00, 48.00 | 58 | 39,00 | 23.00, 56.00 | 57 | 39,00 | 23.00, 53.00 | 1 | 11,00 | 11.00, 11.00 |
| Post-surgery I-QoL | 83 | 85 | 72.00, 94.00 | 25 | 75,00 | 64.00, 86.00 | 58 | 88,00 | 75.00, 97.00 | 57 | 88,00 | 75.00, 97.00 | 1 | 94,00 | 94.00, 94.00 |
| Diff. post- and pre-surgery I-QoL | 83 | 45 | 29.00, 57.00 | 25 | 38,00 | 26.00, 52.00 | 58 | 47,00 | 29.00, 57.00 | 57 | 47,00 | 35.00, 58.00 | 1 | 83,00 | 83.00, 83.00 |
| **Previous radiotherapy** | | | | | | | | | | | | | | | |
| Pre-surgery 24h PWT | 28 | 644,00 | 377.50, 951.50 | 2 | 629,50 | 470.00, 789.00 | 26 | 632,00 | 370.00, 940.00 | 4 | 745,00 | 371.00, 917.50 | 22 | 643,00 | 370.00, 963.00 |
| Post-surgery 24h PWT | 28 | 10,50 | 0.00, 29.00 | 2 | 50,50 | 26.00, 75.00 | 26 | 6,00 | 0.00, 20.00 | 4 | 1,50 | 0.00, 43.50 | 22 | 10,50 | 0.00, 27.00 |
| Diff. post- and pre-surgery 24h PWT | 28 | -634,50 | -916.50, -369.50 | 2 | -579,00 | -763.00, -395.00 | 26 | -632,00 | -893.00, -358.00 | 4 | -743,50 | -874.00, -371.00 | 22 | -613,50 | -963.00, -358.00 |
| Pre-surgery I-QoL | 14 | 34,00 | 15.00, 45.00 | 1 | 34,00 | 34.00, 34.00 | 13 | 34,00 | 15.00, 45.00 | 1 | 17,00 | 17.00, 17.00 | 12 | 35,00 | 14.00, 45.00 |
| Post-surgery I-QoL | 14 | 88,00 | 84.00, 92.00 | 1 | 84,00 | 84.00, 84.00 | 13 | 88,00 | 86.00, 92.00 | 1 | 91,00 | 91.00, 91.00 | 12 | 88,00 | 84.00, 92.50 |
| Diff. post- and pre-surgery I-QoL | 14 | 51,00 | 44.00, 71.00 | 1 | 50,00 | 50.00, 50.00 | 13 | 52,00 | 44.00, 71.00 | 1 | 74,00 | 74.00, 74.00 | 12 | 50,00 | 43.50, 66.50 |
| **No concomittant detrusor overactivity** | | | | | | | | | | | | | | | |
| Pre-surgery 24h PWT | 81 | 474,00 | 282.00, 789.00 | 22 | 494,00 | 325.00, 789.00 | 59 | 454,00 | 225.00, 875.00 | 48 | 471,50 | 225.00, 737.00 | 11 | 350,00 | 171.00, 1200.00 |
| Post-surgery 24h PWT | 81 | 5,00 | 0.00, 21.00 | 22 | 12,00 | 5.00, 25.00 | 59 | 2,50 | 0.00, 17.00 | 48 | 2,50 | 0.00, 18.50 | 11 | 9,00 | 0.00, 17.00 |
| Diff. post- and pre-surgery 24h PWT | 81 | -463,00 | -763.00, -274.00 | 22 | -482,00 | -763.00, -320.00 | 59 | -452,50 | -826.00, -225.00 | 48 | -470,00 | -724.50, -221.50 | 11 | -350,00 | -1200.00, -152.00 |
| Pre-surgery I-QoL | 50 | 39,00 | 23.00, 55.00 | 20 | 36,00 | 25.00, 53.50 | 30 | 42,00 | 20.00, 55.00 | 26 | 40,00 | 20.00, 56.00 | 4 | 46,00 | 28.00, 51.00 |
| Post-surgery I-QoL | 50 | 87,00 | 75.00, 95.00 | 20 | 77,50 | 70.50, 87.00 | 30 | 94,00 | 84.00, 98.00 | 26 | 94,50 | 84.00, 98.00 | 4 | 93,50 | 87.50, 96.50 |
| Diff. post- and pre-surgery I-QoL | 50 | 45,00 | 30.00, 56.00 | 20 | 40,00 | 27.00, 51.00 | 30 | 48,00 | 36.00, 60.00 | 26 | 48,50 | 36.00, 60.00 | 4 | 46,00 | 39.50, 65.50 |
| **Concomittant detrusor overactivity** | | | | | | | | | | | | | | | |
| Pre-surgery 24h PWT | 64 | 449,00 | 303.00, 653.00 | 12 | 432,50 | 286.50, 592.50 | 52 | 484,00 | 304.00, 663.00 | 43 | 395,00 | 282.00, 663.00 | 9 | 630,00 | 529.00, 743.00 |
| Post-surgery 24h PWT | 64 | 10,50 | 0.00, 28.50 | 12 | 0,00 | 0.00, 26.50 | 52 | 13,00 | 0.00, 28.00 | 43 | 11,00 | 0.00, 29.00 | 9 | 19,00 | 4.00, 27.00 |
| Diff. post- and pre-surgery 24h PWT | 64 | -416,50 | -636.00, -263.50 | 12 | -406,00 | -585.00, -202.50 | 52 | -450,00 | -650.00, -281.00 | 43 | -334,00 | -650.00, -246.00 | 9 | -590,00 | -743.00, -523.00 |
| Pre-surgery I-QoL | 39 | 34,00 | 22.00, 49.00 | 5 | 22,00 | 19.00, 23.00 | 34 | 38,50 | 25.00, 52.00 | 27 | 39,00 | 27.00, 53.00 | 7 | 34,00 | 13.00, 44.00 |
| Post-surgery I-QoL | 39 | 82,00 | 69.00, 90.00 | 5 | 56,00 | 52.00, 64.00 | 34 | 84,50 | 71.50, 92.00 | 27 | 83,00 | 70.00, 92.00 | 7 | 88,00 | 75.00, 92.00 |
| Diff. post- and pre-surgery I-QoL | 39 | 45,00 | 29.00, 54.00 | 5 | 33,00 | 26.00, 42.00 | 34 | 46,00 | 30.00, 52.00 | 27 | 45,00 | 26.00, 51.00 | 7 | 59,00 | 43.00, 71.00 |
| **No previous surgery for urinary incontinence** | | | | | | | | | | | | | | | |
| Pre-surgery 24h PWT | 127 | 490,00 | 282.00, 772.00 | 23 | 474,00 | 323.00, 635.00 | 104 | 493,00 | 282.00, 777.00 | 84 | 471,50 | 276.00, 769.50 | 20 | 681,50 | 360.00, 981.50 |
| Post-surgery 24h PWT | 127 | 5,00 | 0.00, 26.00 | 23 | 8,00 | 0.00, 24.00 | 104 | 5,00 | 0.00, 26.00 | 84 | 5,00 | 0.00, 26.00 | 20 | 10,50 | 0.00, 25.50 |
| Diff. post- and pre-surgery 24h PWT | 127 | -477,00 | -747.00, -261.00 | 23 | -458,00 | -635.00, -320.00 | 104 | -480,00 | -772.00, -261.00 | 84 | -456,50 | -730.50, -221.50 | 20 | -639,50 | -968.50, -354.00 |
| Pre-surgery I-QoL | 78 | 35,00 | 22.00, 51.00 | 17 | 27,00 | 19.00, 40.00 | 61 | 40,00 | 22.00, 52.00 | 51 | 39,00 | 22.00, 53.00 | 10 | 39,00 | 15.00, 45.00 |
| Post-surgery I-QoL | 78 | 86,00 | 73.00, 94.00 | 17 | 69,00 | 58.00, 78.00 | 61 | 88,00 | 82.00, 97.00 | 51 | 90,00 | 78.00, 97.00 | 10 | 88,00 | 86.00, 92.00 |
| Diff. post- and pre-surgery I-QoL | 78 | 47,50 | 33.00, 57.00 | 17 | 33,00 | 30.00, 50.00 | 61 | 48,00 | 36.00, 58.00 | 51 | 48,00 | 36.00, 59.00 | 10 | 48,00 | 43.00, 71.00 |
| **Previous surgery for urinary incontinence** | | | | | | | | | | | | | | | |
| Pre-surgery 24h PWT | 28 | 432,50 | 298.50, 700.00 | 12 | 560,00 | 310.00, 840.00 | 16 | 395,00 | 243.00, 534.00 | 13 | 365,00 | 302.00, 534.00 | 3 | 515,00 | 171.00, 529.00 |
| Post-surgery 24h PWT | 28 | 13,00 | 2.50, 25.00 | 12 | 7,50 | 2.50, 27.50 | 16 | 13,00 | 0.00, 23.00 | 13 | 13,00 | 0.00, 23.00 | 3 | 19,00 | 6.00, 27.00 |
| Diff. post- and pre-surgery 24h PWT | 28 | -388,50 | -647.50, -284.00 | 12 | -507,50 | -825.00, -303.50 | 16 | -382,00 | -534.00, -243.00 | 13 | -334,00 | -534.00, -281.00 | 3 | -488,00 | -523.00, -152.00 |
| Pre-surgery I-QoL | 19 | 33,00 | 24.00, 56.00 | 9 | 39,00 | 26.00, 51.00 | 10 | 30,00 | 13.00, 56.00 | 7 | 33,00 | 24.00, 57.00 | 3 | 13,00 | 11.00, 36.00 |
| Post-surgery I-QoL | 19 | 82,00 | 75.00, 90.00 | 9 | 82,00 | 75.00, 88.00 | 10 | 79,50 | 75.00, 94.00 | 7 | 75,00 | 60.00, 90.00 | 3 | 94,00 | 75.00, 95.00 |
| Diff. post- and pre-surgery I-QoL | 19 | 46,00 | 26.00, 60.00 | 9 | 46,00 | 24.00, 56.00 | 10 | 45,00 | 28.00, 62.00 | 7 | 38,00 | 26.00, 48.00 | 3 | 62,00 | 59.00, 83.00 |
| **No previous surgery for urethral/anastomotic stricture** | | | | | | | | | | | | | | | |
| Pre-surgery 24h PWT | 122 | 449,00 | 282.00, 743.00 | 24 | 480,00 | 322.50, 765.00 | 98 | 445,00 | 281.00, 714.00 | 80 | 438,00 | 257.50, 705.00 | 18 | 522,00 | 331.00, 940.00 |
| Post-surgery 24h PWT | 122 | 6,50 | 0.00, 25.00 | 24 | 5,00 | 0.00, 24.50 | 98 | 7,00 | 0.00, 26.00 | 80 | 7,00 | 0.00, 26.00 | 18 | 7,50 | 0.00, 19.00 |
| Diff. post- and pre-surgery 24h PWT | 122 | -435,50 | -714.00, -255.00 | 24 | -443,00 | -725.50, -320.00 | 98 | -441,00 | -714.00, -246.00 | 80 | -423,00 | -678.50, -217.50 | 18 | -505,50 | -940.00, -300.00 |
| Pre-surgery I-QoL | 76 | 34,00 | 22.00, 51.50 | 18 | 27,00 | 23.00, 45.00 | 58 | 36,00 | 20.00, 53.00 | 48 | 37,50 | 22.00, 54.50 | 10 | 34,00 | 13.00, 45.00 |
| Post-surgery I-QoL | 76 | 86,00 | 73.50, 95.00 | 18 | 77,00 | 68.00, 84.00 | 58 | 89,00 | 75.00, 97.00 | 48 | 90,00 | 74.50, 97.00 | 10 | 87,00 | 82.00, 94.00 |
| Diff. post- and pre-surgery I-QoL | 76 | 48,00 | 35.00, 59.00 | 18 | 44,00 | 26.00, 54.00 | 58 | 48,00 | 35.00, 59.00 | 48 | 47,50 | 35.50, 59.00 | 10 | 55,50 | 44.00, 71.00 |
| **Previous surgery for urethral/anastomotic stricture** | | | | | | | | | | | | | | | |
| Pre-surgery 24h PWT | 33 | 618,00 | 332.00, 777.00 | 11 | 474,00 | 323.00, 650.00 | 22 | 656,00 | 332.00, 777.00 | 17 | 561,00 | 304.00, 777.00 | 5 | 767,00 | 656.00, 1000.00 |
| Post-surgery 24h PWT | 33 | 8,00 | 0.00, 22.00 | 11 | 8,00 | 5.00, 28.00 | 22 | 7,00 | 0.00, 20.00 | 17 | 5,00 | 0.00, 10.00 | 5 | 20,00 | 19.00, 35.00 |
| Diff. post- and pre-surgery 24h PWT | 33 | -590,00 | -772.00, -332.00 | 11 | -474,00 | -620.00, -287.00 | 22 | -612,00 | -772.00, -332.00 | 17 | -553,00 | -772.00, -295.00 | 5 | -747,00 | -974.00, -637.00 |
| Pre-surgery I-QoL | 21 | 40,00 | 23.00, 51.00 | 8 | 35,50 | 18.50, 57.50 | 13 | 43,00 | 27.00, 45.00 | 10 | 40,50 | 27.00, 51.00 | 3 | 44,00 | 13.00, 45.00 |
| Post-surgery I-QoL | 21 | 84,00 | 75.00, 89.00 | 8 | 74,00 | 59.50, 87.50 | 13 | 84,00 | 83.00, 91.00 | 10 | 83,50 | 82.00, 91.00 | 3 | 88,00 | 88.00, 92.00 |
| Diff. post- and pre-surgery I-QoL | 21 | 43,00 | 30.00, 52.00 | 8 | 31,50 | 25.50, 42.50 | 13 | 48,00 | 39.00, 57.00 | 10 | 46,50 | 28.00, 57.00 | 3 | 48,00 | 43.00, 75.00 |

**PWT:** Pad weight test
**TP:** Transperineal
**TS:** Transscrotal
**TC:** Transcorporeal cuff placement in patients operated with a transscrotal incision
