# Supplementary material for: Performance and safety of transverse scrotal vs transperineal AUS for PPUI: A retrospective cohort study
Source: BJUI Compass. 2025 May 21;6(5):e70027. doi: 10.1002/bco2.70027 (PMC12094818; doi:10.1002/bco2.70027)
Supplement: Supplementary file 3 — Table S3. Difference in medians regarding changes in 24‐h pad weight test and I‐QoL. There was no difference in medians for the reduction in 24‐h pad weight test between surgical groups when stratifying for previous radiotherapy, detrusor overactivity, surgery for urinary incontinence or surgery for urethral or anastomotic stricture. For patients with no concomitant detrusor overactivity, previous surgery for urinary incontinence or surgery for urethral or anastomotic stricture, we found a higher increase in I‐QoL index after implantation in patients benefitting from a transscrotal approach compared to patients operated with a transperineal approach. Imputed data analysis. [file BCO2-6-e70027-s001.pdf]

Supplemental table 3

Difference in functional and qualitative outcomes for different surgical approaches, stratified analysis. Imputaded data analysis.

	Crude difference of medians								Adjusted difference of medians							
	Diff	95% CIs		p-value	Diff	95% CIs		p-value	Diff	95% CIs		p-value	Diff	95% CIs		p-value
**Previous radiotherapy**	No				Yes				No				Yes			
Difference between post- and pre-surgery 24h PWT																
TS vs TP	29,48	-122,05	181,01	0,701	-13,38	-575,11	548,35	0,960	0,16	-10,63	10,95	0,977	-30,43	-117,88	57,02	0,478
TS without TC vs TP	29,48	-119,59	178,55	0,696	-45,34	-794,21	703,53	0,901	-0,14	-10,86	10,59	0,980	-31,59	-142,63	79,46	0,562
TC vs TP	319,48	209,23	429,73	***0,000***	9,58	-568,83	587,99	0,973	15,19	4,03	26,35	***0,008***	-29,57	-116,00	56,86	0,485
Difference between post- and pre-surgery I-QoL																
TS vs TP	8,76	-4,26	21,78	0,184	-0,36	-33,33	32,61	0,982	8,67	-0,83	18,17	0,073	1,92	-17,49	21,33	0,837
TS without TC vs TP	8,70	-4,22	21,62	0,184	8,98	-32,56	50,52	0,655	8,35	-1,71	18,41	0,103	6,32	-22,53	35,18	0,652
TC vs TP	45,38	33,89	56,87	***0,000***	-2,72	-36,31	30,87	0,865	22,21	12,48	31,93	***0,000***	1,05	-19,12	21,23	0,913
**Detrusor overactivity**	No				Yes				No				Yes			
Difference between post- and pre-surgery 24h PWT																
TS vs TP	9,56	-202,17	221,29	0,929	-63,16	-290,11	163,79	0,580	-10,58	-26,24	5,08	0,181	7,91	-15,80	31,61	0,507
TS without TC vs TP	-3,04	-212,25	206,17	0,977	44,64	-201,10	290,38	0,718	-10,00	-25,94	5,95	0,214	7,89	-15,41	31,19	0,501
TC vs TP	108,36	-479,42	696,14	0,714	-182,66	-410,04	44,72	0,113	-6,93	-28,91	15,05	0,529	10,31	-20,16	40,77	0,501
Difference between post- and pre-surgery I-QoL																
TS vs TP	8,20	-6,38	22,78	0,266	10,06	-9,43	29,55	0,304	11,21	3,25	19,17	***0,006***	10,32	-9,34	29,98	0,296
TS without TC vs TP	6,96	-8,03	21,95	0,357	8,02	-12,13	28,17	0,426	11,31	2,79	19,83	***0,010***	7,58	-12,59	27,74	0,453
TC vs TP	13,92	-9,95	37,79	0,247	22,38	-0,76	45,52	0,058	10,72	-0,05	21,48	0,051	16,38	-5,66	38,42	0,141
**Previous surgery for urinary incontinence**	No				Yes				No				Yes			
Difference between post- and pre-surgery 24h PWT																
TS vs TP	-17,94	-173,55	137,67	0,820	98,64	-243,72	441,00	0,558	-5,55	-23,70	12,61	0,545	4,80	-14,74	24,34	0,617
TS without TC vs TP	-9,54	-164,58	145,50	0,903	146,64	-209,00	502,28	0,403	-5,44	-23,08	12,21	0,542	5,31	-16,11	26,74	0,613
TC vs TP	-133,68	-406,13	138,77	0,333	-7,36	-442,53	427,81	0,972	-1,59	-23,94	20,75	0,888	10,82	-12,74	34,38	0,352
Difference between post- and pre-surgery I-QoL																
TS vs TP	11,52	-2,35	25,39	0,102	2,96	-23,17	29,09	0,816	13,05	0,32	25,78	***0,045***	-2,05	-18,98	14,87	0,802
TS without TC vs TP	10,30	-3,82	24,42	0,151	-1,42	-26,59	23,75	0,908	12,54	-0,01	25,10	***0,050***	-3,56	-20,72	13,59	0,669
TC vs TP	17,90	-2,18	37,98	0,080	17,90	-8,07	43,87	0,167	13,46	0,25	26,67	***0,046***	13,03	-8,94	34,99	0,232
**Previous surgery for urethral/anastomotic stricture**	No				Yes				No				Yes			
Difference between post- and pre-surgery 24h PWT																
TS vs TP	32,10	-145,64	209,84	0,721	-120,72	-364,61	123,17	0,320	-0,05	-18,63	18,54	0,996	-5,92	-24,81	12,96	0,525
TS without TC vs TP	31,52	-160,12	223,16	0,745	-61,72	-352,01	228,57	0,667	0,43	-18,67	19,53	0,965	-9,10	-30,06	11,85	0,375
TC vs TP	5,84	-244,84	256,52	0,963	-255,72	-603,16	91,72	0,143	-0,24	-21,21	20,74	0,982	4,67	-32,22	41,57	0,796
Difference between post- and pre-surgery I-QoL																
TS vs TP	5,18	-5,71	16,07	0,348	14,76	-5,54	35,06	0,145	9,36	0,84	17,88	***0,032***	14,70	-2,53	31,93	0,091
TS without TC vs TP	3,28	-7,64	14,20	0,552	12,88	-9,73	35,49	0,248	8,49	-0,97	17,95	0,078	13,89	-4,29	32,07	0,127
TC vs TP	13,76	-2,52	30,04	0,096	27,40	-8,59	63,39	0,127	10,70	1,16	20,23	***0,028***	17,53	-4,03	39,09	0,106

TP: Transperineal

TS: Transscrotal

TC: Transcorporeal cuff placement in patients operated with a transscrotal incision

PWT: Pad weight test
