# Supplementary material for: Performance and safety of transverse scrotal vs transperineal AUS for PPUI: A retrospective cohort study
Source: BJUI Compass. 2025 May 21;6(5):e70027. doi: 10.1002/bco2.70027 (PMC12094818; doi:10.1002/bco2.70027)
Supplement: Supplementary file 4 — Table S4. Relative risks for serious adverse device events, stratified analysis. Relative risk for serious adverse device events depending on different surgical techniques, stratified for previous radiation therapy, previous surgery for incontinence, previous surgery for urethral/anastomotic stricture and detrusor overactivity. Empty cells show that no patient within the strata had the complication of interest. [file BCO2-6-e70027-s002.pdf]

Supplemental table 4

Relative risks of serious adverse device events, stratified analysis

Empty cells show that no patient within the strata had the complication of interest.

	Previous radiotherapy							
	No				Yes			
	RR	95% CIs		p-value	RR	95% CIs		p-value
**Infection**								
TS including TC vs TP	0,38	0,08	1,78	0,218				
TS excluding TC vs TP	0,38	0,08	1,80	0,223				
TC vs TP								
**Erosion**								
TS including TC vs TP	0,50	0,12	2,14	0,351				
TS excluding TC vs TP	0,51	0,12	2,16	0,358				
TC vs TP								
**Mechanical failure**								
TS including TC vs TP	0,43	0,23	0,79	***0,007***	0,57	0,09	3,75	0,560
TS excluding TC vs TP	0,43	0,24	0,80	***0,008***				
TC vs TP					0,67	0,10	4,35	0,672
**Revision**								
TS including TC vs TP	0,43	0,24	0,77	***0,004***	0,57	0,09	3,75	0,560
TS excluding TC vs TP	0,43	0,24	0,77	***0,005***				
TC vs TP					0,67	0,10	4,35	0,672
**Explantation**								
TS including TC vs TP	0,50	0,19	1,35	0,172	1,14	0,19	6,75	0,883
TS excluding TC vs TP	0,51	0,19	1,36	0,179	1,60	0,21	11,92	0,646
TC vs TP					1,07	0,18	6,44	0,944

	Previous detrusor overactivity							
	No				Yes			
	RR	95% CIs		p-value	RR	95% CIs		p-value
**Infection**								
TS including TC vs TP	0,75	0,07	7,87	0,808	0,09	0,01	0,81	***0,032***
TS excluding TC vs TP	0,94	0,09	9,92	0,961	0,12	0,01	1,06	0,056
TC vs TP								
**Erosion**								
TS including TC vs TP	0,93	0,19	4,50	0,931	1,63	0,21	12,56	0,641
TS excluding TC vs TP	0,71	0,13	3,97	0,694	0,71	0,07	7,32	0,774
TC vs TP	1,79	0,28	11,33	0,538	4,57	0,58	36,25	0,150
**Mechanical failure**								
TS including TC vs TP	0,47	0,21	1,05	0,064	0,35	0,15	0,79	***0,012***
TS excluding TC vs TP	0,41	0,17	1,01	0,053	0,41	0,18	0,94	***0,035***
TC vs TP	0,67	0,21	2,12	0,496	0,16	0,02	1,17	0,071
**Revision**								
TS including TC vs TP	0,47	0,21	1,05	0,064	0,39	0,18	0,86	***0,019***
TS excluding TC vs TP	0,41	0,17	1,01	0,053	0,46	0,20	1,02	0,057
TC vs TP	0,67	0,21	2,12	0,496	0,16	0,02	1,17	0,071
**Explantation**								
TS including TC vs TP	1,00	0,29	3,46	0,994	0,54	0,19	1,57	0,260
TS excluding TC vs TP	0,94	0,26	3,47	0,930	0,27	0,07	1,06	0,061
TC vs TP	1,19	0,23	6,29	0,837	1,43	0,47	4,30	0,526

	Previous surgery for incontinence							
	No				Yes			
	RR	95% CIs		p-value	RR	95% CIs		p-value
**Infection**								
TS including TC vs TP	0,24	0,05	1,14	0,072				
TS excluding TC vs TP	0,32	0,07	1,48	0,143				
TC vs TP								
**Erosion**								
TS including TC vs TP	1,33	0,31	5,67	0,701	1,63	0,17	15,99	0,677
TS excluding TC vs TP	0,79	0,16	3,85	0,768	1,00	0,07	14,34	1,000
TC vs TP	3,11	0,68	14,12	0,142	4,33	0,37	51,29	0,245
**Mechanical failure**								
TS including TC vs TP	0,48	0,25	0,92	***0,026***	0,16	0,02	1,22	0,078
TS excluding TC vs TP	0,47	0,24	0,94	***0,032***	0,20	0,03	1,48	0,116
TC vs TP	0,52	0,20	1,33	0,170				
**Revision**								
TS including TC vs TP	0,51	0,27	0,96	***0,036***	0,14	0,02	0,99	***0,049***
TS excluding TC vs TP	0,50	0,26	0,99	***0,046***	0,17	0,02	1,20	0,075
TC vs TP	0,52	0,20	1,33	0,170				
**Explantation**								
TS including TC vs TP	0,77	0,31	1,94	0,583	0,81	0,13	5,01	0,823
TS excluding TC vs TP	0,57	0,21	1,56	0,272	0,50	0,05	4,86	0,550
TC vs TP	1,45	0,52	4,03	0,477	2,17	0,28	16,76	0,459

	Previous surgery for urethral stricture							
	No				Yes			
	RR	95% CIs		p-value	RR	95% CIs		p-value
**Infection**								
TS including TC vs TP	0,25	0,05	1,19	0,083				
TS excluding TC vs TP	0,33	0,07	1,52	0,154				
TC vs TP								
**Erosion**								
TS including TC vs TP	1,02	0,23	4,53	0,981	2,69	0,35	20,84	0,343
TS excluding TC vs TP	0,49	0,09	2,78	0,419	2,21	0,26	19,08	0,471
TC vs TP	2,92	0,62	13,69	0,175	4,00	0,43	36,92	0,221
**Mechanical failure**								
TS including TC vs TP	0,34	0,18	0,63	***0,001***	0,90	0,25	3,21	0,868
TS excluding TC vs TP	0,35	0,18	0,68	***0,002***	0,74	0,17	3,12	0,678
TC vs TP	0,29	0,09	0,91	***0,034***	1,33	0,29	6,23	0,715
**Revision**								
TS including TC vs TP	0,33	0,18	0,60	***<0,001***	0,90	0,25	3,21	0,868
TS excluding TC vs TP	0,35	0,19	0,65	***0,001***	0,74	0,17	3,12	0,678
TC vs TP	0,27	0,09	0,83	***0,023***	1,33	0,29	6,23	0,715
**Explantation**								
TS including TC vs TP	0,56	0,21	1,48	0,242	1,88	0,45	7,88	0,385
TS excluding TC vs TP	0,39	0,13	1,18	0,096	1,47	0,31	6,95	0,624
TC vs TP	1,17	0,38	3,55	0,786	3,00	0,64	14,02	0,163

TP: Transperineal

TS: Transscrotal

TC: Transcorporeal cuff placement in patients operated with a transscrotal incision
